# Sleep quality and clinical association with sleep disturbance in systemic sclerosis

**DOI:** 10.1186/s41927-023-00346-7

**Published:** 2023-07-21

**Authors:** Nonthaphorn Wongthawa, Apichart So-gnern, Ajanee Mahakkanukrauh, Siraphop Suwannaroj, Chingching Foocharoen

**Affiliations:** 1 Division of Rheumatology, Department of Medicine, Faculty of Medicine, Khon Kaen University, Khon Kaen, 40002 Thailand; 2 Division of Sleep Medicine, Department of Medicine, Faculty of Medicine, Khon Kaen University, Khon Kaen, 40002 Thailand

**Keywords:** Systemic sclerosis, Scleroderma, Sleep quality, Sleep disturbance, Pittsburgh Sleep Quality Index, Depression

## Abstract

**Background:**

Poor sleep quality is a common and potentially debilitating problem in systemic sclerosis (SSc). To date, no data clarifies the potential factors related to poor sleep quality and the clinical associations with sleep disturbance among Thais with SSc—mainly the diffuse cutaneous SSc (dcSSc) subset. We aimed to evaluate sleep quality and identify the clinical association with sleep disturbance among SSc patients.

**Methods:**

A cross-sectional study was conducted between May 2021 and September 2021. Adult SSc patients were enrolled at the Scleroderma Clinic, Khon Kaen University, Thailand. All patients had their neck circumference measured, underwent airway evaluation using the Mallampati classification, had sleep quality assessed using the Pittsburgh Sleep Quality Index (PSQI), and the Berlin and Patient Health Questionnaire-9 completed. In addition, the clinical association with poor sleep quality (or sleep disturbance) was investigated using the PSQI.

**Results:**

A total of 88 patients were enrolled. Forty-eight (54.6%) patients experienced poor sleep quality (95%CI 43.6–65.2). Digital ulcers and dyspepsia were associated with poor sleep quality as per a logistic regression (OR 10.73: 95%CI 1.09–106.15 and 4.60: 95%CI 1.01–20.89), respectively. Overall pain—evaluated using the visual analog scale (VAS)—was positively correlated with the PSQI score (Rho 0.2586; *p* = 0.02).

**Conclusion:**

Around half of the SSc patients reported poor sleep quality, and the significantly associated factors were digital ulcers and dyspepsia. The PSQI scores positively correlated with overall pain as evaluated by VAS. With early assessment and treatment of digital ulcers, stomach symptoms, and pain control, sleep problems might be reduced among SSc patients.

**Supplementary Information:**

The online version contains supplementary material available at 10.1186/s41927-023-00346-7.

## Key messages

1. Poor sleep quality is a common sleep problem in systemic sclerosis.

2. Disease activity, especially vascular, gastrointestinal involvement, and pain, affect sleep quality.

**Supplementary Information:**

The online version contains supplementary material available at 10.1186/s41927-023-00346-7.

## Introduction

Systemic sclerosis (SSc) is a connective tissue disease characterized by several clinical features, and skin tightness is the classic clinical feature. The principal pathogenic mechanisms include fibrosis, vasculopathy, and immune dysregulation. The greater the degree of organ fibrosis and vasculopathy, the greater the effect on the quality of life and sleep. Difficulty sleeping is a common and potentially debilitating problem in SSc [1, 2], with 76% of patients reporting difficulty sleeping at least sometimes and 59% reporting moderate to severe impact on daily function [2].

Sleep quality has been reported in SSc patients according to studies that used the Pittsburgh Sleep Quality Index (PSQI) [3], the Medical Outcomes Study Sleep measure (MOS-sleep scale) [4], the Patient-Reported Outcome Measurement Information System (PROMIS) Sleep Disturbance Scale (short form) [5]. The PSQI is a commonly used questionnaire that quantifies sleep quality disturbance. It is relevant to adult and pediatric sleep medicine research and clinical practice. The original PSQI validation was performed with mixed-age healthy controls—individuals with major depression and sleep clinic patients [6]. Research has confirmed the reliability and validity of the PSQI in various populations, including patients with cancer and other medical conditions [7]. The PSQI is a simple assessment tool that costs much less than standard polysomnography. The PSQI evaluates seven aspects of sleep problems over the past month, including sleep latency, duration, habitual sleep efficiency, sleep disturbances, sleeping medication use, daytime dysfunction, and subjective sleep quality. The final score ranges between 0 and 21. The higher the score, the more serious the sleep problems.

Sariyildiz et al. [3] evaluated sleep quality using the PSQI in 48 SSc patients and compared them to 42 healthy control subjects. Sariyildiz et al. [3] found that SSc patients had a significantly higher PSQI than the healthy controls. Sleep disturbance in SSc patients was significantly correlated with pain, depression, fatigue, functional status, and the SF-36 physical score [3]. Their study, however, did not evaluate the correlation between the SSc subset and sleep quality.

A recent study among SSc using the PSQI quantified the prevalence of sleep disturbance at 73.3% [7]. The study, however, included patients with SSc overlap with other connective tissue diseases and sine scleroderma, for which the clinical characteristics differ from those with pure SSc. Nearly half of the patients in the study had the limited cutaneous SSc (lcSSc) subset, which is less severe than the diffuse cutaneous SSc (dcSSc) subset, which is more common in the Thai population [8] along with a higher prevalence of anti-Scl70 positive [9].

Clarity regarding the factors potentially related to sleep problems in SSc is limited due to (a) the small number of published studies, (b) the relatively small sample sizes of existing studies, and (c) the lack of critical data on critical factors (i.e., airway patency) that could be related to poor sleep quality in SSc [7]. We, therefore, aimed to evaluate sleep quality and the clinical factors associated with sleep disturbance in Thai SSc. If clinical associations with sleep disturbance are confirmed, the findings should guide interventions to improve sleep quality and quality of life.

## Method

### Study design

From May to September 2021, we conducted an analytical cross-sectional study. Participants were recruited from the Scleroderma Clinic at Srinagarind Hospital, Khon Kaen University, Khon Kaen, Thailand. Before participating in the study, enrollees signed an informed consent form. After enrollment, the subjects received routine clinical care for SSc; then, they completed a self-administered PSQI, the Berlin questionnaire, a pain score, and the patient health questionnaire-9 (PHQ-9).

### Population

We recruited SSc patients over 18 years of age who could speak, read, and understand Thai. All of the patients had a diagnosis of SSc based on the American College of Rheumatology criteria, and/or they fulfilled the classification criteria for systemic sclerosis as per the ACR/EULAR 2013 [10]. The SSc was classified as either limited cutaneous SSc (lcSSc) or diffuse cutaneous SSc (dcSSc) as per LeRoy et al. [11] We excluded SSc patients who (a) had overlap with other connective tissue diseases; (b) were pregnant or lactating or bedridden; (c) could not care for themselves; (d) had evidence of active malignant disease, uncontrolled medical problems; (e) needed hospitalization; (f) presented active infection; (g) had a current neurological or psychiatric problem rendering them unable to understand the questionnaire; (h) had an event that might disrupt sleep within one week before enrollment (i.e., long trip, overseas travel, or nightshift work); or, (g) received anxiolytic, hypnotic, or sedative agents within 2 weeks before enrollment.

### Clinical evaluation

Demographic data from the medical records were reviewed, including age, sex, body mass index (BMI), and comorbid disease. The clinical assessments included (a) tests for vasculopathy (Raynaud’s phenomenon, ischemic ulcer, digital gangrene, and telangiectasia); (b) skin and soft tissue (calcinosis cutis, salt, and pepper appearance); (c) the severity of skin involvement using modified Rodnan Skin Score (mRSS) [12]; (d) the presence of tendon friction rub, hand deformities, arthritis, weakness, cardiopulmonary symptoms (i.e., cough, chest pain, pulmonary fibrosis, and pulmonary arterial hypertension (PAH)); and, (e) gastrointestinal symptoms (esophageal, stomach, or intestinal symptoms such as dysphagia, heartburn, dyspepsia, or constipation). The airway was evaluated per the Mallampati classification [13] and neck circumference [14]. Pain symptoms were assessed using the visual analog scale (VAS) (Supplementary file 1). The PSQI [15] and the Berlin Questionnaire in Thai version [16] were used to evaluate sleep quality and disordered sleep breathing, respectively. Depression was assessed using the patient health questionnaire-9 (PHQ-9) in Thai version [17]. All patients completed the questionnaire by themselves or their relatives and/or research assistants if they could not read or write.

### Operational definitions

The onset of SSc was defined by the first time any non-Raynaud’s phenomenon SSc symptoms occurred. Disease duration was calculated as the interval between disease onset and the time at the last data collection. Pulmonary fibrosis was defined when HRCT revealed interstitial fibrosis. PAH was characterized by a mean pulmonary arterial pressure > 20 mmHg and a pulmonary capillary wedge pressure < 15 mmHg from right heart catheterization [18]. Esophageal involvement was defined when any esophageal symptoms of SSc presented—viz., esophageal dysphagia, heartburn, or reflux symptoms. Heartburn was a burning sensation in the neck or back to the sternum that worsened after eating or lying down [19]. On the other hand, reflux was the sensation of gastric contents returning to the mouth or hypopharynx [19]. Stomach involvement was indicated by dyspepsia, early satiety, or vomiting. Intestinal involvement was characterized by diarrhea, bloating, malabsorption, constipation, ileus, or pseudo-intestinal obstruction. Overall pain was the pain that occurred anywhere in the body as assessed using a visual analog scale. Poor sleep quality was indicated by the total score ≥ 6 as calculated from the seven components of the PSQI [15, 20]. Depressive disorder was indicated when the Thai version of the PHQ-9 score was ≥ 9 [17].

### Sample size

According to the literature review, the sample size of 83 was based on an alpha of 0.05, an absolute precision of 0.90, and a sleep disturbance prevalence of 68–73.3% [7, 21]. The study's secondary objective sought to determine the factors associated with poor sleep quality in SSc. According to the literature review, ten poor sleep-quality SSc patients would be needed to study at least six factors associated with poor sleep quality [3, 7, 21, 22]. Thus, at least 88 SSc patients were required for the current study.

### Statistical analysis

Clinical characteristics were categorized into dichotomous, polytomous, or continuous variables. The prevalence of sleep disturbance was calculated with its 95% confidence interval (95%CI). The odds ratio with 95%CI was tested to determine the clinical association with poor sleep quality or sleep disturbance in SSc patients. Variables significantly associated with poor sleep quality from the literature review and variables with *p* < 0.1 were chosen and entered into a logistic regression model. Pearson’s or Spearman Rank correlation was used to evaluate the correlation between the PSQI and clinical parameters. *P*-values < 0.05 were considered statistically significant. The data were analyzed using STATA version 16.0 (StataCorp., College Station, TX, USA).

## Results

A total of 88 patients were enrolled. The majority were female (57 cases; 64.8%) and had the dcSSc subset (56 cases; 63.6%). The respective mean age and median disease duration was 61.3 ± 9.9 years and 7.4 years (IQR 3.6–11.1). The patient demographic data are presented in Table 1.

**Table 1:** Demographic data

Variable	Total *n* = 88
Female (%)	57 (64.8)
Age (years); mean ± SD	61.3 ± 9.9
Age at onset (years); mean ± SD	52.6 ± 11.9
Disease duration (year); median (IQR)	7.4 (3.6–11.1)
BMI (kg/m^2^); mean ± SD	21.1 ± 4.1
SSc subsets	
lcSSc (%)	32 (36.4)
dcSSc (%)	56 (63.6)
mRSS (points); median (IQR)	2 (0–8)
Vasculopathy	
Active Raynaud’s phenomenon (%)	25 (28.4)
Active digital ulcer (%)	8 (9.0)
Digital gangrene (%)	1 (1.1)
Telangiectasia (%)	36 (40.9)
Musculoskeletal symptoms	
Tendon rub (%)	4 (4.6)
Hand deformity (%)	34 (38.6)
Synovitis (%)	9 (10.23)
Gastrointestinal symptoms	
Esophageal involvement (%)	39 (44.3)
Dysphagia	11
Heartburn	11
Both dysphagia and heartburn	17
Stomach involvement* (%)	16 (18.2)
Intestinal involvement (%)	15 (17.1)
Pulmonary symptoms	
Cough (%)	22 (25.0)
Chest pain (%)	1 (1.1)
ILD (%)	52 (59.1)
Renal crisis (%)	1 (1.1)
Pulmonary hypertension (%)	11 (12.5)
Pain	
Joint pain (%)	25 (28.4)
Muscle pain (%)	16 (18.2)
Generalize pain (%)	4 (4.6)
Low back pain (%)	17 (19.3)
Tooth pain (%)	5 (5.7)
Headache (%)	7 (7.9)
Depressive disorder (%)	11 (12.5)
Neck circumference (cm); mean ± SD	32.8 ± 2.7
Mallampati classification	
I (%)	19 (21.6)
II (%)	11 (12.5)
III (%)	25 (28.4)
IV (%)	33 (37.5)

*IQR* interquartile range, *lcSSc* limited cutaneous systemic sclerosis, *dcSSc* diffuse cutaneous systemic sclerosis, *ILD* Interstitial lung disease, *mRSS* modified Rodnan skin score

^*^all dyspepsia

### Prevalence of poor sleep quality

Based on the PSQI questionnaire, 48 SSc were defined as having poor sleep quality with a prevalence of 54.6% (95% CI 43.6–65.2). The mean PSQI score was 6.52 ± 3.59.

### Clinical association with poor sleep quality

According to the univariable analysis, patients with poor sleep quality had more frequent digital ulcer(s), dyspepsia, low back pain, and depressive disorder than those with normal sleep quality (Table 2).

**Table 2:** Clinical difference between the patients with poor sleep quality and normal sleep quality

Variable	Normal sleep quality *n* = 40	Poor sleep quality *n* = 48	OR (95%CI)	*P*-value
Female (%)	23 (57.5)	34 (70.8)	1.79 (0.68–4.78)	0.19
Age (years); median (IQR)	60.9 (57.5–64.4)	60.7 (57.5–63.9)	-	0.89
Age at onset (years); median (IQR)	53.6 (49.9–57.3)	50.8 (46.9–54.7)	-	0.30
BMI (kg/m^2^); mean ± SD	20.9 ± 4.1	21.2 ± 4.1	-	0.78
SSc subsets				
lcSSc (%)	16 (40.0)	16 (33.3)	1	NA
dcSSc (%)	24 (60.0)	32 (66.7)	1.33 (0.50–3.48)	NA
mRSS (points); median (IQR)	2(0–6)	2(0–10)	-	0.53
Vasculopathy				
Active Raynaud’s phenomenon (%)	8 (20.0)	17 (35.4)	2.19 (0.75–6.72)	0.11
Active Digital ulcer (s) (%)	1 (2.5)	7 (14.58)	6.66 (0.78–307.58)	0.045^a^
Digital gangrene (%)	1 (2.5)	0 (0.0)	NA	NA
Telangiectasia (%)	16 (40)	20 (41.7)	1.07 (0.42–2.75)	0.87
Musculoskeletal symptoms				
Tendon rub (%)	1 (2.5)	3 (6.3)	2.6 (0.19–139.91)	0.4
Hand deformity (%)	13 (32.5)	21 (43.8)	1.65 (0.62–4.26)	0.28
Synovitis (%)	4 (10.0)	5 (10.42)	1.05 (0.21–5.68)	0.95
Cardiopulmonary symptoms				
Cough (%)	9 (22.5)	13 (27.1)	1.23 (0.42–3.77)	0.67
Chest pain (%)	0 (0.0)	1 (2.1)	NA	NA
Gastrointestinal symptoms				
Esophageal involvement (%)	21 (52.5)	18 (37.5)	0.54 (0.21–1.38)	0.15
Heartburn (%)	15 (37.5)	13 (27.1)	0.62 (0.23–1.68)	0.30
Stomach involvement^b^ (%)	3 (7.5)	13 (27.1)	4.58 (1.10–26.74)	0.02^a^
Intestinal involvement (%)	5 (12.5)	10 (20.8)	1.84 (0.51–7.52)	0.30
ILD (%)	26 (65)	26 (54.2)	0.63 (0.24–1.63)	0.30
Renal crisis (%)	1 (2.5)	0 (0.0)	NA	NA
Pulmonary hypertension (%)	7 (17.5)	4 (8.3)	0.43 (0.09–1.87)	0.19
Pain				
Joint pain (%)	12 (30.0)	13 (27.08)	0.84 (0.29–2.36)	0.70
Muscle pain (%)	7 (17.5)	9 (18.8)	1.08 (0.32–3.84)	0.89
Generalize pain (%)	1 (2.5)	3 (6.25)	2.59 (0.19–139.51)	0.40
Low back pain (%)	4 (10.0)	13 (27.1)	3.34 (0.90–15.25)	0.04^a^
Tooth pain (%)	2 (5.0)	3 (6.3)	1.26 (0.14–15.81)	0.80
Headache (%)	1 (2.5)	6 (12.5)	5.56 (0.62–262.35)	0.09
Depressive disorder (%)	1 (2.5)	10 (20.8)	10.26 (1.32–456.49)	0.01^a^
Neck circumference (cm); mean ± SD	33.0 ± 2.6	32.6 ± 2.7	-	0.56
Mallampati classification				
I (%)	7 (17.5)	12 (25.0)	1	
II (%)	6 (15.0)	5 (10.4)	0.49 (0.11–2.20)	0.35
III (%)	10 (25.0)	15 (31.3)	0.88 (0.26–2.99)	0.83
IV (%)	17 (42.5)	16 (33.3)	0.55 (0.17–1.74)	0.31

*IQR* interquartile range, *OR* odds ratio, *95%CI* 95% confidence interval, *lcSSc* limited cutaneous systemic sclerosis, *dcSSc* diffuse cutaneous systemic sclerosis, *ILD* Interstitial lung disease, *mRSS* modified Rodnan skin score

^a^statistically significant, NA not available due to statistical limitation (zero number of cases in one cell)

^b^all dyspepsia

The factors associated with poor sleep quality according to the logistic regression were digital ulcer and stomach involvement (dyspepsia) with an adjusted OR of 10.73 (95%CI 1.09–106.15) and 4.60 (95%CI 1.01–20.89), respectively (Table 3). The clinical association between poor sleep quality and depressive disorder suggested by the logistic regression was only a trend, according to the follow-up statistical analysis (Table 3).

**Table 3:** Logistic regression analysis of factors associated with poor sleep quality

Variable	Crude OR (95% CI)	*P*-value	Adjusted OR (95% CI)	*P*-value
Digital ulcer	6.66 (0.78–307.58)	0.045	10.73 (1.09–106.15)	0.042^*^
Stomach involvement^a^	4.58 (1.10–26.74)	0.02	4.60 (1.01–20.89)	0.048^*^
Heartburn	0.62 (0.23–1.68)	0.30	0.43 (0.14–1.31)	0.139
Cough	1.23 (0.42–3.77)	0.67	0.69 (0.21–2.32)	0.553
Low back pain	3.34 (0.90–15.25)	0.04	2.20 (0.55–8.74)	0.263
Depressive disorder	10.26 (1.32–456.49)	0.01	9.07 (1.00–82.56)	0.050

*OR* odds ratio, *95%CI* 95% confidence interval

^*^statistical significant

^a^all were dyspepsia

There was a significant positive correlation between the PSQI and pain using the VAS with a *rho* of 0.2586 (*p* = 0.02); however, there was no correlation between the PSQI and age, disease duration, mRSS, BMI, and neck circumference (data not shown).

## Discussion

Sleep problems, particularly poor sleep quality, are frequently presented in SSc patients. The prevalence of sleep disturbance among SSc patients is between 40 and 90%, depending on the method of sleep quality assessment, the definition of poor sleep quality, and the target population [3–5, 7, 21, 22]. Our study included 88 SSc patients evaluating sleep quality using the PSQI and found that around half of the SSc patients had poor sleep quality based on the questionnaire with a mean PSQI score of 6.5. Compared to a previous study on sleep quality assessment using the PSQI, Sariyildiz et al. [3] found that the mean PSQI among Turkish SSc was 7.5 ± 3.0, and the prevalence of poor sleep quality was 68.8%. By comparison, Figueiredo et al. [7] reported the respective mean of PSQI among Brazilian SSc, and the prevalence of poor sleep quality was 9.5 ± 4.3 and 73.3%.

Thai SSc patients seem to have less poor sleep quality than White races despite a higher proportion of dcSSc, the more severe form among Thais and associated with a poor prognosis [8]. Differences in the study population, culture, patient attitudes, self-awareness, and prevalence of depression might explain the findings. The presence of depression is associated with isolation, unemployment, and loss of quality of life and has been closely linked to sleep disturbance in the general population [23, 24]. Based on previous studies, depression is a factor associated with poor sleep quality in SSc [3, 7, 21], but not in the present study. We found that depressive mood was less commonly reported among Thai SSc patients (12.5%) than was reported in the studies by Sariyildiz et al. [3] (62.5%) and Figueiredo et al. [7] (56.6%). The low numbers of depression among Thais might explaine the lower prevalence of poor sleep quality among Thai SSc than the earlier reports [3, 7, 22]. A trend in the association between poor sleep disturbance and depressive disorder in our logistic regression analysis (*p* = 0.05) suggests that depression is still likely to be a significant factor in poor sleep quality in SSc, despite the low prevalence of depression in our population compared to previous studies. According to our findings and literature review, the prevalence of poor sleep quality is probably mainly affected by patient psychological status, particularly depression, rather than disease severity.

Dyspepsia was associated with poor sleep quality in our SSc patients. The finding is similar to a previous study in which the link between sleep disturbance and gastrointestinal involvement was reported; [4, 5, 7, 20], except that most were associated with esophageal symptoms (gastroesophageal reflux disease; GERD) rather than dyspepsia [7, 21]. Although data are lacking regarding the direct association between dyspepsia and poor sleep quality in SSc, a significant association has been confirmed between both factors in the general population [25–27].

The relationship between poor sleep quality and dyspepsia is complex. Dyspeptic symptoms could interfere with sleep, as abdominal pain may delay the onset or interrupt sleep continuation [25, 27]. Alternatively, poor sleep quality could lead to increased symptom expression of dyspepsia [25]. Unfortunately, our study method cannot confirm whether dyspepsia is the cause or the effect of poor sleep quality. We, therefore, suggest a further longitudinal study of sleep quality to define the correct relationship between dyspepsia and sleep quality. Nevertheless, once gastrointestinal symptoms, especially dyspepsia, are reported, lifestyle modifications (acidic food avoidance) and intensive medical treatment for controlling the symptoms should be used to improve patient sleep quality and/or quality of life.

Digital ulcers were also associated with poor sleep quality among Thai SSc. Vasculopathy causing digital ulcers in SSc results in severe ischemic pain, especially at night, also causing difficulty sleeping [28–31]. Hence, it is unsurprising that a digital ulcer affects sleep quality. According to the nature of the disease, vasculopathy is more often revealed in patients who live in cold climates. Notwithstanding, no study on Caucasians in Europe, North America, or Australia has reported on the association despite having a higher prevalence of digital ulcers than Thais (50–55% in Caucasians vs. 20% in Thais) [32, 33]. The finding of digital ulcers could pique interest in sleep quality even in warm climates and prompt management for early healing of ulcers to reduce the risk of poor sleep quality.

In our results, esophageal involvement, particularly GERD or heartburn, was not a factor related to poor sleep quality despite its strong association with sleep disturbance in previous studies [4, 7, 21]. In addition to chronic cough, which represents interstitial lung disease (ILD), GERD might also disturb the quality of life and sleep [1, 4, 21, 34], but it did not affect sleep quality in our study despite the high numbers of patients with ILD. This was an unexpected finding, and the explanation is unclear. Since we did not evaluate the severity of heartburn and cough in those patients, we are reluctant to explain the findings due to a mild degree of disease severity.

Upper airway anatomical balance evaluated using the Mallampati classification, and neck circumference did not affect sleep quality in our SSc patients; however, these factors increased the prevalence of obstructive sleep apnea (OSA) among SSc patients in previous studies [35, 36]. OSA is a sleep-related breathing disorder in which the main feature is partial or complete airway obstructions during sleep. A high Mallampati score (less airway structure visualization) and/or high neck circumference can lead to airway blockages at night, snoring, and OSA [14]. Since poor sleep quality and sleep-related breathing disorders are different sleep problems, it is possible that the factors associated with both sleep problems are not necessarily the same. The Mallampati classification and neck circumference might not be appropriate for evaluating sleep quality in SSc.

According to the literature review, the prevalence of poor sleep quality and its association varied according to differences in definition, methods of sleep quality assessment, and study populations. A comparison of the prevalence and associated factors of poor sleep quality in SSc patients are summarized in Table 4.

**Table 4:** Comparison of the prevalence and associated factors of poor sleep quality in SSc patients

Authors	Year	Country	N	Method of sleep quality assessment	Definition of poor sleep quality	Prevalence of poor sleep quality (%)	Factor association
Our study	2021	Thailand	88 (32 lcSSc; 36.4%, 56 dcSSc; 63.6%)	PSQI with mean ± SD of 6.52 ± 3.59	PSQI ≥ 6	54.6% (95%CI 43.6–65.2)	• Digital ulcer (OR 10.73) • Stomach involvement^a^ (OR 4.60)
Carandina et al. [20]	2021	Italy	20 (16 lcSSc; 80%)	PSQI with median of 8 (IQR 7–10)	PSQI > 5	90%	• Cardiovascular autonomic control (*p* = 0.03) • Pain (*p* = 0.03) • Depressive symptoms (*p* < 0.01)
Figueiredo et al. [7]	2020	Brazil	60 (29 lcSSc; 48.3%)	PSQI with mean ± SD of 9.5 ± 4.3	PSQI ≥ 7^b^	73.3%	• Esophageal involvement (*p* = 0.03) • High disease severity (*p* = 0.01) • Depressive mood (*p* = 0.002)
Horsley-Silva et al. [19]	2019	USA	287 (184 lcSSc; 64%)	PSQI in moderate to severe compared to mild heartburn mean ± SD of 9.16 ± 3.98 and 6.75 ± 3.49	PSQI > 5	68%	GERD symptoms (OR 2.53)
Sariyildiz et al. [3]	2013	Turkey	48 (lcSSc 45.5%)	PSQI with mean ± SD of 7.5 ± 3.0	PSQI ≥ 6	68.8%	• Generalized pain • Fatigue • Depressive symptoms • Functional status • Physical score of the SF-36 (*p* < 0.001)
Milette et al. [5]	2013	Canada	397 (98 dcSSc; 24.7%)	PROMIS (sleep disturbance scale short form) with mean ± SD of 22.8 ± 8.0	ND	ND	Pearson correlation with PROMIS score • Gastrointestinal symptoms (*p* = 0.001) • Pain (*p* < 0.001) • Pruritus (*p* = 0.024)
Frech et al. [4]	2011	USA	180 (90 lcSSc; 50.9%)	MOS-Sleep measure with mean ± SD of 7.1 ± 1.73 h of sleep a night	Quantity of sleep is scored as the average hours slept per night (0–11 h) report worse score on 4 of 6 scales (except for snoring and sleep quantity)	• 41% for high-ceiling effects for snoring • 59% for shortness of breath scales	• Worsening pain and dyspnea over the past 1 month • Reflux scale of the UCLA SCTC GIT 2.0, • CESD • FACIT-Fatigue

*ND* No data, *USA* United States of America, *ESS* Epworth Sleepiness Scale, *IQR* Interquartile range, *SE* Sleep efficiency, *REM* of rapid eye movement, *SWS* Slow wave sleep, *PLMI* Periodic leg movement Index, *RLS* Restless legs syndrome, *MOS-Sleep scale* Medical Outcomes Study sleep measure, *SLP-9* 9-item sleep problem Index, *AHI* Apnea–hypopnea index, *OSA* Obstructive sleep apnea, *SDB* Sleep-disordered breathing, *ODI* Oxygen desaturation index, *SpO2* pulse oximetry, *UCLA SCTC GIT 2.0* University of California at Los Angeles Scleroderma Clinical trial Consortium Gastrointestinal Tract 2.0, *CESD* Center for Epidemiologic Studies Short Depression scale, *FACIT-Fatigue* Functional Assessment of Chronic Illness Therapy-Fatigue

^a^All were dyspepsia

^b^Cut point for Brazilian Portuguese version

The limitations of the study include a) a cross-sectional design that increases the recall bias on the questionnaire, albeit the results provide insight into the actual situation vis-à-vis sleep quality evaluation in daily practice and obviate the need for an in-patient hospitalization evaluation; b) the lack of physiologic testing in patients with reported dyspepsia, so we cannot conclude whether the patients had pathologic or functional dyspepsia; and, c) the lack of recorded disease severity, so we cannot provide an association between disease severity and sleep quality in Thai SSc. Nevertheless, we included all the clinical parameters that might affect sleep quality in the analysis, so most of the critical SSc features were assessed.

The strengths of this study were that: a) we included a large study population and sample size calculation, so there is a high level of confidence in the estimated prevalence of poor sleep quality among Thai SSc patients; and, b) the parameters that can affect sleep quality were evaluated, including neck circumference and Mallampati classification. Our findings may help evaluate sleep quality and provide insights into how to improve the daily practice care of SSc patients.

## Conclusion

The prevalence of poor sleep quality was approximately 55% among Thai SSc. Sleep quality was impacted by vasculopathy, gastrointestinal involvement, and overall pain. Earlier evaluation and treatment of digital ulcers, stomach symptoms, and pain control may improve SSc patients' sleep quality.

## Supplementary Information

**Additional file 1. **

## Data Availability

The datasets used and/or analyzed during the current study are available from the corresponding author upon reasonable request.
