# Supplementary material for: Sleep quality and clinical association with sleep disturbance in systemic sclerosis
Source: BMC Rheumatol. 2023 Jul 21;7:21. doi: 10.1186/s41927-023-00346-7 (PMC10360221; doi:10.1186/s41927-023-00346-7)
Supplement: Supplementary file 1 — Additional file 1. [file 41927_2023_346_MOESM1_ESM.pdf]

Subject ID ☐☐☐☐

Code ☐☐☐☐-☐☐ **Date of visit** ☐☐-☐☐-☐☐☐☐

In the last week, do you have any pain in your body and how severity of the pain?

Please circle the score that reflects how much of your pain

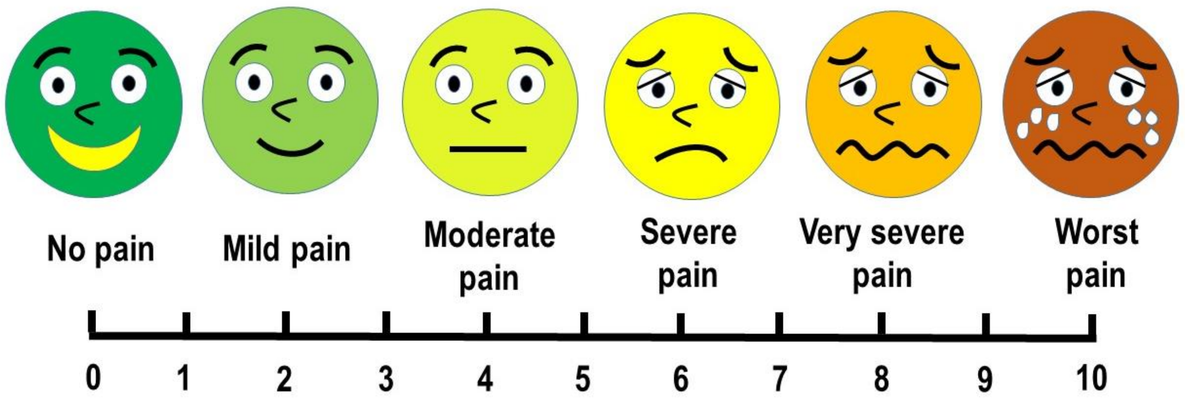

Please write ✓ down in ☐ that compatible with your answers (can tick more than 1 box)

You have any symptoms of the following:

☐ **ulcer pain at tip of fingers or toes**		☐ **joint pain**
☐ **muscle pain**	☐ **diffuse pain**	☐ **back pain**
☐ **toothache**	☐ **headache**	☐ **chest pain**
☐ **chest discomfort**	☐ **frank pain**	☐ **stomach pain or discomfort**
☐ **heart burn**	☐ **other pain please define..**	
